# Exploring the Inhibition of Quercetin on Acetylcholinesterase by Multispectroscopic and In Silico Approaches and Evaluation of Its Neuroprotective Effects on PC12 Cells

**DOI:** 10.3390/molecules27227971

**Published:** 2022-11-17

**Authors:** Yijing Liao, Xi Mai, Xiaqing Wu, Xing Hu, Xiaoqiao Luo, Guowen Zhang

**Affiliations:** 1 State Key Laboratory of Food Science and Technology, Nanchang University, Nanchang 330047, China; 2 School of Pharmaceutical Science, Nanchang University, Nanchang 330006, China; 3 Faculty of Life Science & Medicine, Northwest University, Xi’an 710069, China

**Keywords:** acetylcholinesterase, quercetin, inhibitory mechanism, computational docking, oxidative stress

## Abstract

This study investigated the inhibitory mechanism of quercetin in acetylcholinesterase (AChE) and its neuroprotective effects on β-amyloid_25–35_-induced oxidative stress injury in PC12 cells. Quercetin inhibited AChE in a reversible mixed manner with an IC_50_ of 4.59 ± 0.27 µM. The binding constant of quercetin with AChE at 25 °C was (5.52 ± 0.05) × 10^4^ L mol^−1^. Hydrogen bonding and van der Waals forces were the main interactions in forming the stable quercetin–AChE complex. Computational docking revealed that quercetin was dominant at the peripheral aromatic site in AChE and induced enzymatic allosterism; meanwhile, it extended deep into the active center of AChE and destabilized the hydrogen bond network, which caused the constriction of the gorge entrance and prevented the substrate from entering the enzyme, thus resulting in the inhibition of AChE. Molecular dynamics (MD) simulation emphasized the stability of the quercetin–AChE complex and corroborated the previous findings. Interestingly, a combination of galantamine hydrobromide and quercetin exhibited the synergistic inhibition effect by binding to different active sites of AChE. In a β-amyloid_25–35_-induced oxidative stress injury model in PC12 cells, quercetin exerted neuroprotective effects by increasing the glutathione level and reducing the malondialdehyde content and reactive oxygen species levels. These findings may provide novel insights into the development and application of quercetin in the dietary treatment of Alzheimer’s disease.

## 1. Introduction

Alzheimer’s disease (AD) is a neurodegenerative disease known to cause dementia [1]. In 2020, 55 million patients with dementia were reported worldwide. It is predicted that by 2050, the number of patients will be nearly triple. The increasing incidence of AD has caused a huge financial burden on individuals and society. The annual cost of dementia in the world is known to exceed 1.3 trillion US dollars [2]. Loss of cognitive function, memory loss, and decreased ability to live a normal life in AD patients severely deteriorate the quality of life of these patients and eventually lead to disability and death [3]. AD is rapidly becoming one of the most expensive, lethal, and burdensome diseases in the 21st century [4]. Similar to other slowly progressive diseases, the prevention of AD depends on the early understanding of its pathogenesis. In recent years, researchers have provided comprehensive information on the cytopathological, biochemical, and genetic basis of the disease as well as biomarkers and neuroimaging patterns [5]. The etiology of AD includes an aggregation of tau (τ) protein, impaired cholinergic neurotransmission, deposition of β-amyloid protein (Aβ), oxidative stress, and neuroinflammation [6]. Acetylcholine (ACh) is a central excitatory neurotransmitter that exerts a critical effect on nerve impulse transmission and memory. Acetylcholinesterase (AChE) is a key enzyme in biological nerve conduction that can rapidly hydrolyze ACh into acetate and choline and terminate ACh-mediated synaptic transmission in the central nervous system with high catalytic efficiency [7]. The “Cholinergic hypothesis” states that loss of cholinergic nerve cells and increased activity of AChE in the basal forebrain of patients with AD lead to decreased levels of ACh in the brain and accelerated the deposition of Aβ. Therefore, inhibition of AChE is considered a promising method to treat AD [8]. Currently, most of the drugs used to treat AD clinically, such as donepezil, galantamine, and rivastigmine, are AChE inhibitors. Although these drugs are known to exert positive effects on the symptomatic treatment of patients with mild to moderate AD, they are ineffective in delaying disease development, and their long-term use produces severe dose-dependent side effects [9].

Oxidative stress (OS) is an important part of several hypotheses of the pathogenesis of AD (amyloid cascade, intracellular neurofibrillary tangles, and metal ions hypothesis). It is a crucial factor in promoting the occurrence and development of AD. Several in vivo and in vitro studies have reported that the Aβ peptide can increase reactive oxygen species (ROS) levels while inducing OS [10].

Due to the complex pathogenic mechanisms that underlie AD, increasing attention has been paid to developing multitarget-directed ligands (MTDLs) to treat AD effectively. Natural ingredients exert an important effect on the treatment of neurodegenerative disorders. In addition, secondary metabolites have been used to discover small-molecule drugs due to their unique chemical characteristics and biological activities [11]. Therefore, it is highly significant to identify natural compounds with antioxidant and AChE inhibitory activities to prevent and treat AD.

**Figure 1:**
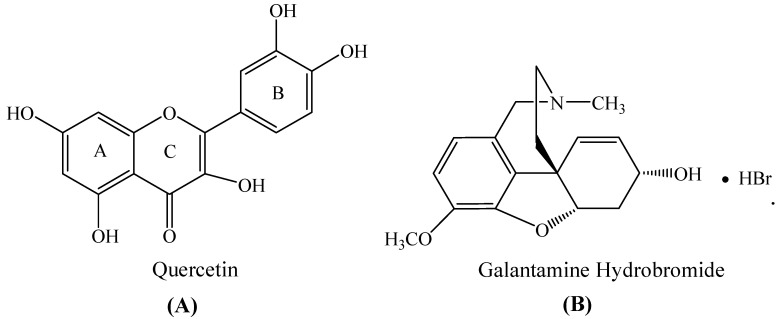
Structure of quercetin (**A**) and galantamine hydrobromide (**B**).

Increasing evidence shows that flavonoids exert their neuroprotective effects by maintaining the quality and quantity of the neurons in key brain areas. It prevents or delays the occurrence and development of age-related neurodegenerative diseases [12,13]. Quercetin (3,3′,4′,5,7-pentahydroxyflavone, Figure 1A) is a dietary flavonoid that exists within numerous plants, fruits, vegetables, and nuts in the form of glycosides and exerts potential nutritional and medicinal values [14]. Quercetin has been commercially used as a functional food and produces potential neuroprotective, antitumor, anti-inflammatory, and cardioprotective properties without significant cytotoxicity [15]. Research on the function of quercetin in anti-AD and improving cognitive and neuroprotection has gradually increased [16]. Quercetin can regulate the expression of thioredoxin and maintain the association of thioredoxin with apoptosis signal-regulating kinase 1 (ASK1) to mediate its neuroprotective function and improve cognitive dysfunction during aging [17]. Low doses of quercetin protect the primary neurons from Aβ_1–42_-induced toxicity by regulating oxidative stress [18]. Quercetin inhibits AChE in a dose-dependent manner and significantly reduces the levels of AChE within the hippocampal neuron homogenate and increases the synaptic concentration of ACh, thus improving the cognitive output of animals [19]. Studies have reported that quercetin is an effective AChE inhibitor; however, the detailed and in-depth mechanism of quercetin-inhibiting AChE has not been reported.

The combined medication can increase the curative effects of therapeutic drugs and reduce their side effects. Multidrug therapy has long been used for medical purposes, such as hypertension, AIDS, and neurodegenerative diseases [20]. Abdel-Diam et al. reported that curcumin and quercetin could synergistically alleviate the subacute diazinon-induced liver and brain inflammation and oxidative nerve injury in albino rats [21]. Our group reported that a combination of vitexin and acarbose exhibited a strong synergistic inhibition of α-glucosidase associated with diabetes [22]. Furthermore, a combination of apigenin and orlistat could synergistically inhibit pancreatic lipase activity related to obesity [23]. Galantamine hydrobromide (Figure 1B) is a competitive reversible AChE inhibitor, isolated from several plant sources such as *Galanthus nivalis* and *Lycoris*. Furthermore, it is one of the approved drugs for the clinical treatment of AD. Galantamine easily penetrates the blood–brain barrier (BBB), inhibits AChE in the cerebral cortex, and has a good improvement effect in patients with AD; however, its efficacy is relatively weak, so it is only suitable for the early treatment of mild and moderate AD [24]. Considering the complexity of the pathophysiology and different symptoms of AD, it is difficult for a single class of drugs to solve the related problems caused by cognitive dysfunction in adults. Therefore, it is practically significant and clinically valuable to study the inhibitory effect and mechanism of a combination of quercetin and galantamine in AChE.

Multispectral techniques have been widely used to study the inhibitory activity and mechanism of natural active components in key metabolic enzymes [25]. Computer technology has been successfully used to predict the detailed binding of ligand receptors, and molecular dynamics (MD) simulation has attracted wide attention in exploring the binding mechanism and conformational behavior of proteins and their potential inhibitors [26]. In the present study, we used multispectral methods, including circular dichroism (CD), fluorescence spectroscopy, and UV–vis absorption spectroscopy, to determine binding profiles, inhibitory kinetics/activity, and the effect of quercetin on the conformation of AChE. The structure-based molecular docking was utilized to predict the binding sites, bonding mode, and the major amino acid residues for the interaction between quercetin and AChE. The structural flexibility and dynamic stability of AChE in the presence of quercetin were investigated with MD simulation studies. The effect and mechanism of a combination of quercetin and galantamine on AChE inhibition were evaluated. To study the neuroprotective effects of quercetin on oxidative stress injury, the AD model of PC12 cells induced by Aβ_25–35_ was established. The mechanism of the inhibitory effect of quercetin on AChE was deeply explored. Subsequently, the anti-AD mechanism of quercetin based on AChE inhibition and oxidative stress damage protection was clarified. The findings of this study will provide new information on the investigation and development of quercetin as a functional food component and a new AChE inhibitor in the prevention and treatment of AD.

## 2. Results and Discussion

### 2.1. Inhibitory Effect of Quercetin on AChE

**Figure 2:**
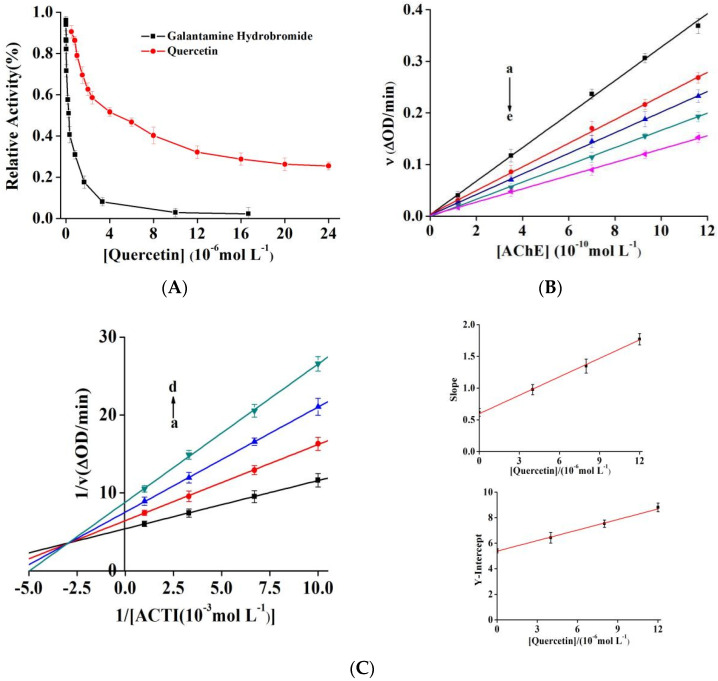

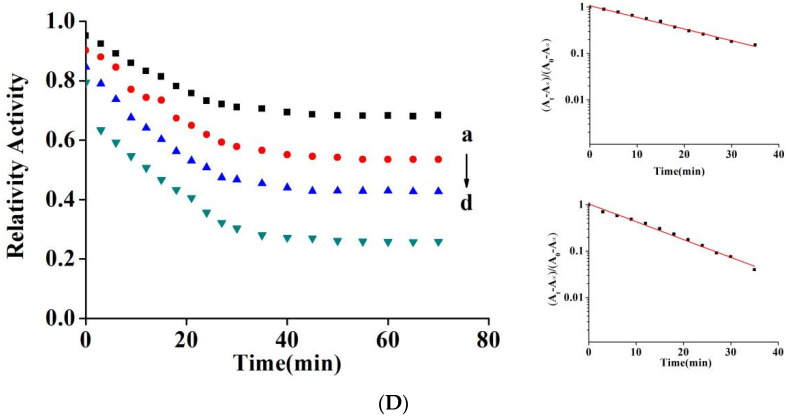
(**A**) Effect of quercetin and galantamine hydrobromide on the activity of AChE at 25 °C (pH 7.6); *c*(AChE) = 0.57 nM, *c*(ATCI) = 0.25 mM. (**B**) Plots of *v* versus (AChE). *c*(ATCI) = 0.25 mM and *c*(quercetin) = 0, 2.0, 3.2, 4.0, and 8.0 µM for curves a → e, respectively. (**C**) Lineweaver-Burk curve of quercetin, *c*(AChE) = 0.57 nM; *c*(quercetin) = 0, 4.0, 8.0, and 12.0 µM for curves a → d. (**D**) Time course for the relative activity of AChE in the presence of quercetin at the concentrations of 1.5, 3.0, 6.0, and 15.0 μM for curves a → d, respectively. *c*(AChE) = 0.57 nM and *c*(ATCI) = 0.25 mM. Semilogarithmic plot analysis for quercetin at 1.5 μM (the upper-right panel) and 15.0 μM (the lower-right panel), and the slope of the curves suggests the inactivation rate constants.

As shown in Figure 2A, quercetin and galantamine hydrobromide significantly inhibited the activity of AChE and the IC_50_ values were 4.59 ± 0.27 and 0.29 ± 0.02 µM, respectively. Jung [27] and Khan [28] reported IC_50_ values of quercetin of 19.8 and 353.86 µM, respectively. Our experimental values were close to the former, both in order of micromolar range. However, it was quite distinct from the latter, which could be due to differences in enzyme concentration, enzyme type, and detection method [29]. With a continuous increase in the quercetin concentration, the relative enzymatic activity of AChE gradually decreased and was stable at approximately 25%. Compared to galantamine, quercetin showed slightly lower inhibitory activity and could not completely inhibit AChE. Furthermore, it was considered a potential bioactivity compound that could prevent both β-amyloidosis and tau hyperphosphorylation [16]. Quercetin demonstrated a considerably lower IC_50_ value than taxifolin (54.01 ± 1.58 µM, saturated bond between C2 and C3) [30], indicating that the double bond at positions C2 and C3 of the C-ring in phenylchromone played an important role in inhibiting the activity of AChE [31,32]. The inhibitory activity of quercitrin (IC_50_ = 94.5 µM), the product of C3-OH replaced by C3-O-Rhamnoside, was significantly lower than that of quercetin. This finding could be ascribed to the dramatic increase in the steric hindrance of quercitrin, which was unsuitable for entering the active site of AChE. In addition, quercetin glycosylation decreased AChE affinity, thus reducing inhibitory activity [32,33].

### 2.2. Inhibition Kinetics of Quercetin

Quantitative inhibitors were added to the reaction solution, and the enzyme activity was measured with its different concentrations. Next, we drew plots for the enzymatic reaction rate as a function of [AChE] to investigate the reversibility of quercetin inhibition (Figure 2B). The straight line at each inhibitor concentration passed through the origin, with an increase in the concentration of quercetin; the slope of the fitting straight line decreased continuously, indicating that quercetin cannot effectively inactivate AChE completely; it only reduced its catalytic rate, making it a typical reversible inhibitor [34].

Four types of reversible inhibition exist: competitive, noncompetitive, anticompetitive, and mixed manner. Using the Lineweaver–Burk method, the double reciprocal curves of enzymatic reaction rate and substrate concentration were drawn (Figure 2C). The fitting lines of quercetin with different concentrations intersect in the second quadrant. With an increase in the quercetin content, *K*_m_ increased gradually while *V*_m_ decreased, indicating the mixed inhibitory type, similar to the function of baicalein in xanthine oxidase [35]. The equations usually describe such mixed inhibition (Equations (S2)–(S4)).

The secondary plots of the slopes and y-intercept versus quercetin showed good linear relationships (*r* = 0.9990 and *r* = 0.9984, respectively), indicating just one or one type of binding site in AChE. According to the equations, the calculated *K*_i_ value was (6.17 ± 0.18) µM, and the *αK*_i_ value was (19.53 ± 0.65) µM. It was evident that *αK*_i_ was greater than *K*_i_, indicating that quercetin can interact with free AChE or the AChE–ATCI complex, and the binding effect with free AChE was stronger [36].

### 2.3. Inactivation Mechanics of Quercetin on AChE

Relative activity curves of AChE were monitored at different quercetin concentrations for 70 min to acquire rate constants and inactivation kinetics. As shown in Figure 2D, quercetin exerted an inhibitory effect on AChE in a dose-dependent manner. Quercetin induced a sharp decrease in AChE activity in the first 30 min, followed by a slight decrease and then balance after 40 min, and remained unchanged after 60 min. The semi-logarithmic diagrams of quercetin (all concentrations) showed good linearity, indicating that quercetin-induced inactivation was a first-order, single-phase process. The equation ΔΔG° = −*R.T.* ln *k* was used to calculate the transition free energy change (ΔΔ*G*°), and the variation in ΔΔ*G*° may inactivate the enzyme [25]. When quercetin concentration was increased from 1.5 µM to 15 µM, the inactivation rate constant (*k*) increased from 4.18 ± 0.06 × 10^−4^ s^−1^ to 6.41 ± 0.05 × 10^−4^ s^−1^ and ΔΔ*G*° decreased from 19.28 to 18.22 kJ mol^−1^ s^−1^ (Table 1).

### 2.4. Fluorescence Quenching of AChE by Quercetin

**Figure 3:**
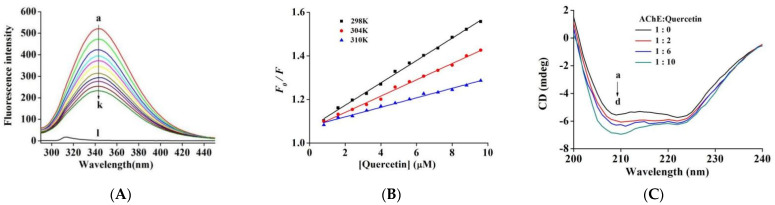
(**A**) Fluorescence spectra of AChE in the presence of quercetin at varying concentrations (pH 7.6, *T* = 25 °C). *c*(AChE) = 0.17 µM, *c*(quercetin) = 0, 0.8, 1.6, 2.4, 3.2, 4.0, 4.8, 5.6, 6.4, 7.2, and 8.0 μM (curves a → k), respectively. Curve l depicts the emission spectrum of quercetin alone. (**B**) The fluorescence quenching curve of quercetin on AChE at 25 °C, 31 °C, and 37 °C. (**C**) The CD spectra of AChE in the presence of quercetin, *c*(AChE) = 0.75 µM; the molar ratios of quercetin to AChE were 0:1, 2:1, 6:1 and 10:1 for curves a → d, respectively.

To further investigate the interplay between quercetin and AChE, a fluorescence quenching analysis was performed to examine the binding affinity and thermodynamic parameters [37]. Following excitation at 280 nm, the maximal fluorescence emission peak was recorded at 343 nm for AChE. Because quercetin did not show fluorescence under the same conditions, it will not cause interference (Figure S1). According to Figure 3A, the fluorescence intensity of AChE was continuously quenched by successive addition of quercetin without a distinct peak shift, implying an interaction between quercetin and AChE.

The Stern–Volmer equation (Equation (S5)) was used to assess fluorescence intensities at different temperatures and elucidate the quercetin-induced AChE fluorescence quenching mechanism.

*F*_0_/*F*_c_ exhibited favorable linearity with quercetin, showing that quercetin has only a single fluorescence quenching type for AChE (Figure 3B). The quenching constant *K*_SV_ (Table 2) was reduced at 25, 31, and 37 °C. Furthermore, the corresponding *K*_q_ values of magnitude order 10^12^ L mol^−1^ s^−1^ were significantly higher than the maximum diffusion collision quenching rate constant of 2.0 × 10^10^ L mol^−1^ s^−1^. These results suggested that quercetin formed a ground-state complex by combining with AChE and induced AChE endogenous fluorescence quenching in the static quenching way [34].

Furthermore, we used the equation (Equation (S6)) to determine the binding site quantity (*n*) and binding constant (*K*_a_) of the AChE-quercetin complex [34]. At 25 to 37 °C temperatures, the *K*_a_ value (Table 2) decreased from 5.52 × 10^4^ L mol^−1^ to 2.05 × 10^4^ L mol^−1^, consistent with the trend of *K*_SV_ value, which further confirmed the static quenching process of quercetin-induced fluorescence quenching. The *K*_a_ value achieved a 10^4^ L mol^−1^ magnitude order, indicating the moderate binding affinity between quercetin and AChE. The values of *n* approached one, indicating the presence of one binding site class for quercetin in AChE, which was in agreement with the results of the inhibition kinetics analyses.

Altogether, small-molecule–biomacromolecule interactions mostly include hydrophobic interactions, electrostatic interactions, van der Waals forces, and hydrogen bonding. The related thermodynamic parameters can be used to determine the main types of interactions. Quercetin forms a static complex with AChE, and the thermodynamic parameters of enthalpy change (∆*H*°), Gibbs free energy (∆*G*°), and entropy change (∆*S*°) can be determined from the van’t Hoff equation (Equations (S7)–(S8)). As shown in Table 2, ∆*G*° was negative, indicating the spontaneous interaction between quercetin and AChE. Furthermore, the negative Δ*S*° and Δ*H*° values indicated exothermic binding, and the primary forces in complex formation were van der Waals interactions and hydrogen bonding [23].

### 2.5. CD Spectra

The activity of quercetin was investigated following the secondary structure of the AChE through CD spectroscopy. As shown in Figure 3C, the spectra of AChE showed two negative bands at 208 nm and 222 nm, which were mainly contributed by α-helical structure [38]. In a system with fixed AChE concentration, quercetin with different molar ratios to AChE was added and incubated for 3 min, and the intensity of both bands increased in a concentration-dependent manner. This illustrated significant alterations of the secondary structure of the protein and indicated the formation of the AChE–quercetin complex, resulting in altered helical stability. Specific secondary structure levels were acquired through an online project. Free AChE was composed of ~34.45% α-helix, ~18.62% β-sheet, ~19.18% β-turn, and ~28.02% random coils that were consistent with the data (~33% α-helix, ~23% β-sheets, ~17% β-turn, and ~27% random coils) reported by Manavalan et al. [39]. As the quercetin-to-AChE molar ratio increased between 0:1 and 10:1, concentrations of α-helix and β-turn elevated to 41.51% and 23.14%, respectively. In contrast, those of random coil and β-sheet declined to 20.19% and 15.16%, respectively (Table 3). As deduced by the increase in α-helix levels, AChE displays a more compact structure. As noted from fluorescence studies, the current results might be due to the formation of hydrogen bonds and alteration in the microenvironment of amino acid residues in AChE while making a complex with quercetin. The conformational changes in AChE hindered the substrate from reaching the active site, thereby reducing the catalytic performance of AChE. Similar results were recorded with epicatechin gallate on xanthine oxidase by Zhu et al. [36].

### 2.6. Molecular Docking

The molecular docking technique allows for visualizing binding sites and conformations for ligands and receptors and explores their interaction mechanism [40]. For the first time, Sussman et al. reported the three-dimensional structure of AChE from Torpedo californica (*Tc*AChE) using X-ray crystal diffraction. Furthermore, most of the binding studies between ligands and AChE were based on the crystal structure of *Tc*AChE [41]. However, some studies have shown that donepezil binds to rhAChE in a conformation significantly different from *Tc*AChE, indicating that hAChE is more accurate for use in drug-binding research [42]. Therefore, we selected human AChE (PDB ID: 4EY7) to dock with quercetin to further predict the interaction force type, the binding site, and the binding mode.

The structure of AChE consists of three core-binding regions: the peripheral aromatic site (PAS), the catalytic active site (CAS), and the gorge. CAS was on the gorge bottom, which contained the catalytic triad Ser203, His447, and Glu334. In addition, the middle gorge connects CAS and PAS and extends to the outside of the enzyme, with a length of about 20 Å and a width of about 5 Å. The inner surface mainly contains aromatic amino acids (Phe295, Phe338, and Tyr337). The PAS in the gorge entrance is largely constituted by five residues, namely, Asp74, Tyr72, Tyr124, Tyr341, and Trp286 [43].

**Figure 4:**
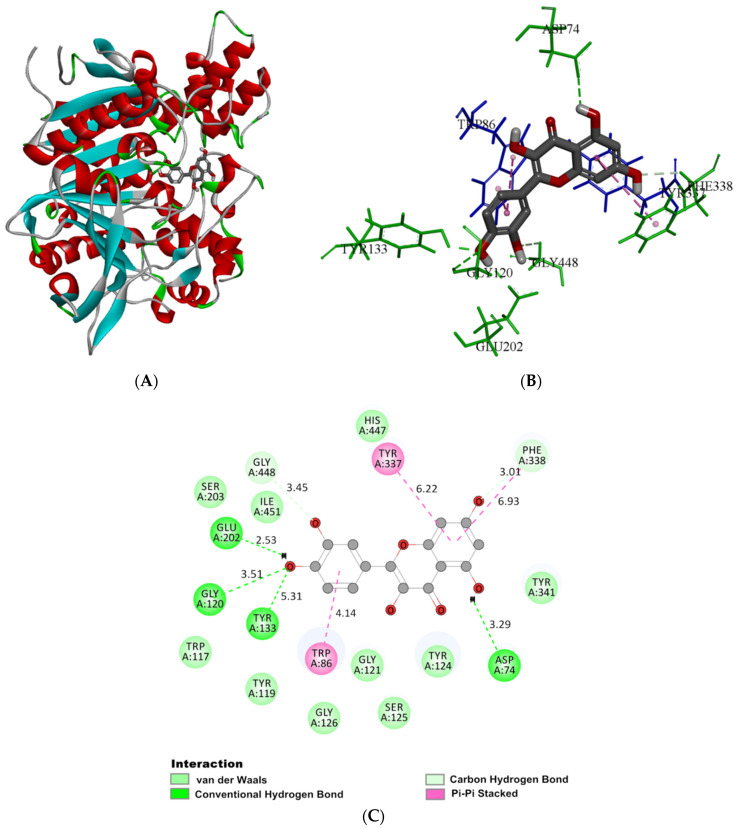
(**A**) The 3D ribbon model of AChE (4EY7) docked with the optimal pose of quercetin. (**B**) The binding area of quercetin in AChE, with only the key residues shown. (**C**) The 2D schematic diagram of the main amino acid residues interacting with quercetin in AChE. The interaction is indicated in different colors.

Molecular docking was adopted for visualizing the interaction of quercetin with AChE. In order to determine the accuracy of the docking, the co-crystallized ligand donepezil was redocked into the active site of AChE (Figure S2). The root mean square deviation (RMSD) between the docking and original co-crystallized pose was 0.844 Å. According to Figure 4A, quercetin was predicted to cross into the active site of AChE, with the minimum binding energy of −45.11 kJ mol^−1^, which was consistent with the value (−8.8 kcal mol^−1^) reported by Islam [44] and the thermodynamic parameters in fluorescence analysis. Figure 4B,C demonstrated the type and intensity of the interaction of quercetin with major amino acid residues within the AChE binding region. Quercetin formed four hydrogen bonds with AChE. The C5-hydroxyhydrogen on the A-ring in phenylchromone generated the hydrogen bond together with oxygen on Asp74 (PAS) residue, and its bond length was 3.29 Å. Hydroxyl hydrogen at the 4′-position of the B ring formed hydrogen bonds with Glu202, and hydroxyl oxygen formed hydrogen bonds with Gly120 and Tyr133, with bond lengths of 2.53, 3.51, and 5.31 Å, respectively. The residue Glu202 was adjacent to the active site of Ser203 residue. Kryger reported that a hydrogen bond network, constructed of Glu202 and Glu450, and Tyr133, was involved in stabilizing the catalytic triad structure of AChE [45]. Quercetin had a hydrogen bonding with Glu202 and Tyr133, which might affect the hydrogen bond network, thus, destroying the stable structure of the AchE active center and reducing its catalytic activity. The parent nucleus of the quercetin had a π-π stacking with Trp86 (choline-binding site), Tyr337, and Phe338, suppressing the action of AchE by inhibiting the induction of the aromatic inner surface of the gorge. The Trp117, Tyr119, Gly121, Tyr124, Ser125, Gly126, Ser203, Tyr341, His447, and Ile451 residues encircled the whole quercetin molecule with van der Waals forces. The C7 hydroxyl oxygen reacted with Phe338, and the C3′ hydroxyl oxygen reacted with Gly448 to form two carbon-hydrogen bonds. All of these residues extended from PAS to CAS at the active site and showed interaction with quercetin to inhibit the AChE effect. The PAS site was associated with allosteric regulation of AChE, and quercetin possibly changed this allosteric structure and constricted the entrance, thus preventing the substrate–enzyme active site combination [46]. Islam [44] indicated that there were 10 hydrogen bonds between quercetin and AChE, forming a higher number of hydrogen bonds than conventional chemical drugs. Khan [28] showed that quercetin binds to AChE with six hydrogen bonds. The number of hydrogen bonds between quercetin and AChE was various, possibly due to different docking methods and different sources of AChE crystal structure. However, conformational changes induced by quercetin of AChE have not been extensively discussed.

Furthermore, the PAS site possibly has a critical effect in accelerating the deposition and aggregation of β-amyloid peptides, indicating that the interaction between quercetin and PAS might reduce nerve injury caused by aggregation of Aβ-amyloid protein [47]. The result was consistent with that of Sabogal-Guaqueta, who demonstrated that quercetin significantly reduced extracellular Aβ-amyloid deposition in cerebral brain regions in a 3xTg-AD mouse model [16].

### 2.7. MD Simulation

The binding of small-molecule ligands to biological macromolecules is a microscopic process that occurs in a very short time. The related parameters of MD can better evaluate the stability and conformational changes of a protein after binding with the ligand and can be used to fill the unpredictable details of experimental methods to further explore its mechanism of action [48]. Herein, MD simulation was performed to explain the structure together with the behavior of AChE and the quercetin–AChE complex.

**Figure 5:**
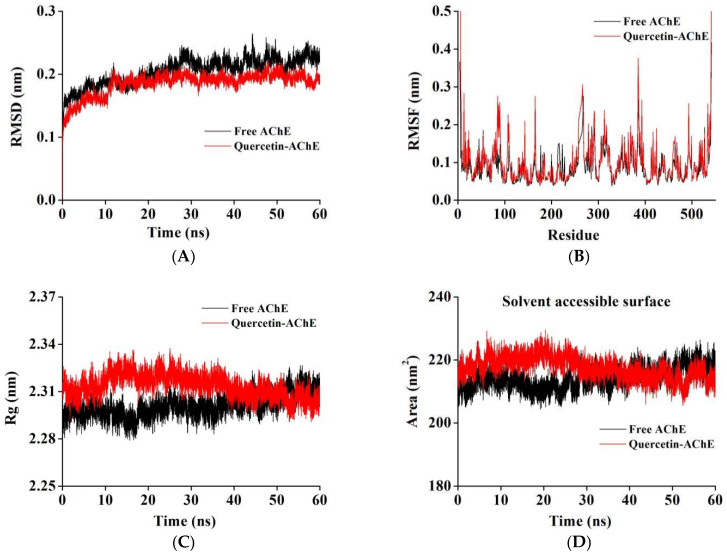
MD simulation of quercetin with AChE for 60 ns. The RMSD (**A**) and RMSF (**B**) plots, Rg values, (**C**) and solvent accessible surface area (**D**) of the quercetin–AChE complex and free AChE backbone.

The root mean square deviation (RMSD) was used to evaluate the degree of deviation of the protein from the original structure, which could explain the stability of the complex after quercetin was bound to AChE in the simulation process [26]. According to Figure 5A, the RMSD values for AChE and the complex gradually increased during 0–12 ns and reached a relatively stable equilibrium after 12 ns with little fluctuation. The RMSD values of the free AChE and AChE–quercetin complex were finally stabilized at approximately 0.21 and 0.19 nm, respectively, indicating that the complex could have a more stable structure than the free AChE due to interactions such as hydrogen bonding [49].

The root mean square fluctuation (RMSF) reflects the flexibility of amino acid residues, which is helpful in deeply understanding the local conformational changes of protein side chains in a simulated time. As shown in Figure 5B, the RMSF values between the amino acid residues of AChE and the AChE–quercetin complex were not significantly different; however, the local amino acid residues, including residues 60–130, 200–230, and 270–340 showed fluctuations, and the amino acid residues in these regions were in agreement with the main amino acid residues that interact with quercetin during MD. It can be inferred that these amino acids with significant fluctuations could actively participate in the binding process between AChE and quercetin and form a stable complex by adjusting their own conformations [50].

The dependence of the radius of rotation (Rg) and solvent-accessible surface area (SASA) on simulation time provides information on the behavior of ligands in enzyme binding pockets. Rg can be determined to illustrate protein compactness, and SASA reflects the surface changes of the system in contact with solvents. As shown in Figure 5C,D, compared to free AChE, the Rg and SASA values of the complex system displayed the same trend, which increased slightly at first and then decreased and stabilized after 40 ns simulation. The Rg and SASA values of the stabilized complex were slightly lower. These results indicated that the interaction between quercetin and AChE might increase the surface hydrophobicity of the enzyme, making the structure more compact and inducing the complex to exist in the binding cavity of the enzyme with a more stable conformation [36,51].

### 2.8. Combined Inhibition Analysis

**Figure 6:**
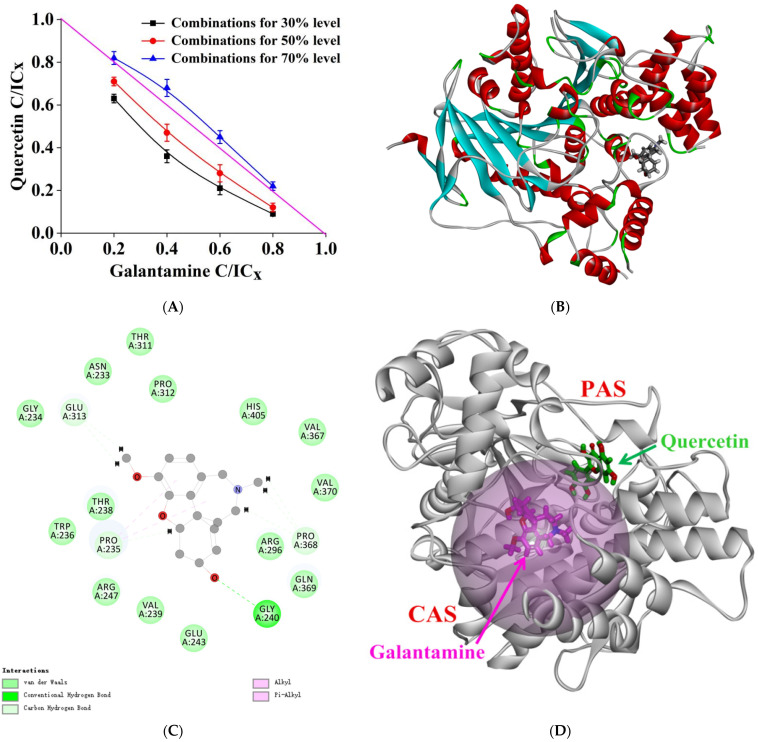
(**A**) Isoboles of additivity for the combination of quercetin and galanthamine hydrobromide at 30%, 50%, and 70% inhibition levels. The magenta straight line stands for an additive isobole with a CI value of 1 in the horizontal and vertical coordinates, which means that any dose point on the line has an additive effect in the combination of two inhibitors. (**B**) The 3D ribbon model of AChE docked with galanthamine. (**C**) The 2D schematic diagram of galanthamine interacting with AChE in the presence of quercetin. (**D**) The 3D interaction pattern of quercetin and galanthamine with AChE. Green and magenta represent quercetin and galanthamine, respectively.

The isoboles of quercetin and galantamine hydrobromide combined inhibition on AChE are shown in Figure 6A. At 30% and 50% inhibition levels, the isoboles were concave and the CI values corresponding to all data on the graph were less than 0.9, indicating that the combination of quercetin and galantamine hydrobromide showed a synergistic inhibition in AChE, which was stronger at 30% inhibition. However, the CI value was between 1 and 1.1 at the 70% inhibition level, meaning that the combination showed an additive effect [22].

Molecular docking was performed to further explain the mechanism of the synergistic effect between quercetin and galantamine hydrobromide. Galantamine is a competitive reversible inhibitor that binds to the CAS, choline site, and acyl site of the active pocket of AChE [24]. As shown in Figure 6B,C, when interacting with AChE in the presence of quercetin, galantamine formed a hydrogen bond with Gly240 residue. There were also van der Waals forces and hydrophobic interactions between galantamine and other amino acid residues. We found that quercetin is a mixed inhibitor that forms hydrogen bonds with residues Glu202 and Tyr133 in the hydrogen bond network and interacts with amino acid residues at PAS. The molecular docking of quercetin and galantamine with AChE showed that galantamine acted on the CAS at the bottom of the gorge. In contrast, quercetin was largely bound to PAS at the entrance. Both acted simultaneously on the enzyme activity center to exert a synergistic inhibitory effect (Figure 6D). The combined inhibition of AChE by quercetin and galantamine primarily showed a synergistic effect. A combination of quercetin and galantamine resulted in an identical inhibition activity at the decreased concentration and reduced adverse effects. This result provides a theoretical basis for developing quercetin as a functional food factor or clinical adjuvant.

### 2.9. Quercetin Cytotoxicity in PC12 Cells

**Figure 7:**
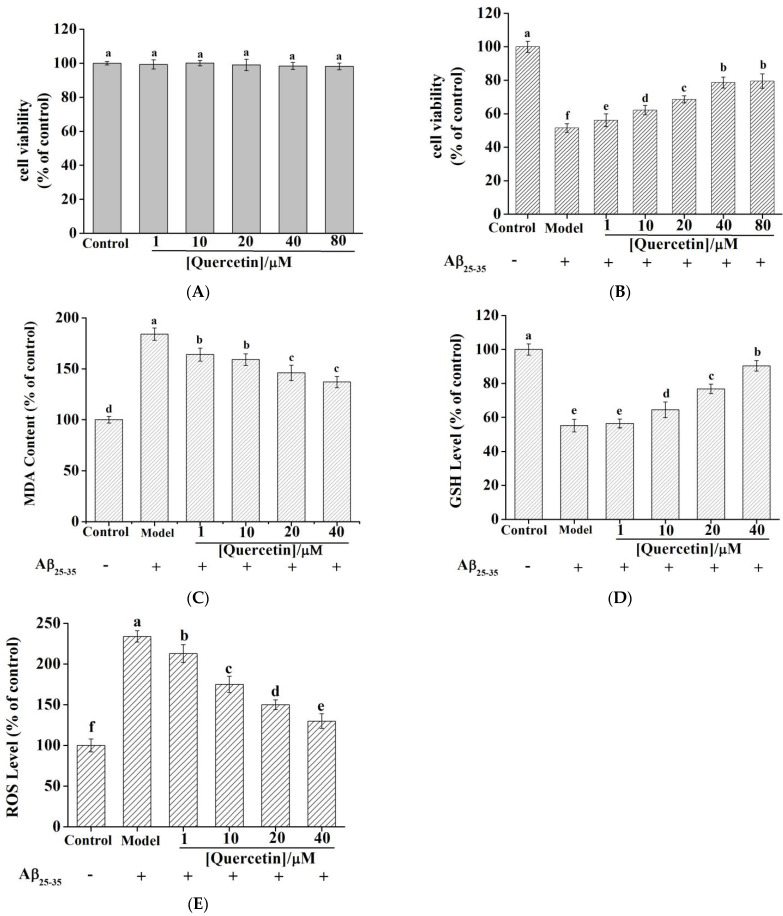
(**A**) The cytotoxicity of quercetin in PC12 cells. The same letter “a” indicates no significant difference (*p* > 0.05). (**B**) Effect of quercetin on the cell viability in Aβ_25−35_—induced PC12 cells. Effect of quercetin on the Aβ_25−35_—induced oxidative stress in PC12 cells: MDA content (**C**) and GSH level (**D**). (**E**) Effect of quercetin on Aβ_25–35_—induced ROS generation in PC12 cells. All data are presented as the mean ± SEM of six independent experiments. In Figure 7A–E, the different superscript letters “a–f” mean significant statistical difference (*p* < 0.05), and the same letters indicate no significant difference (*p* > 0.05).

An MTT assay was performed to evaluate the cytotoxicity of quercetin on PC12 cells. Figure 7A demonstrates that the viability of PC12 cells remained unchanged with different concentrations of quercetin (*n* = 6, *p* > 0.05), indicating that quercetin exerted no toxic effect on PC12 cells at 1–80 µM.

### 2.10. Quercetin Ameliorated Aβ_25–35_—Induced Toxicity in PC12 Cell

PC12 cell injury induced by Aβ_25–35_ is a common and ideal cell model to study AD in vitro [52]. As shown in Figure 7B, the model group achieved (51.43 ± 2.61)% cell viability of the control group following a 24 h treatment using 20 µM Aβ_25–35_ (*n* = 6, *p* < 0.01), indicating that Aβ_25–35_ successfully caused injury to PC12 cells. However, pretreatment of PC12 cells with 1, 10, 20, 40, and 80 µM quercetin increased cell viability to (56.06 ± 3.82)%, (62.14 ± 2.79)%, (68.58 ± 2.12)%, (78.61 ± 3.26)%, and (79.48 ± 4.32)% of the control group, respectively. Relative to the model group, quercetin promoted the viability of PC12 cells in a dose-dependent manner, suggesting a neuroprotective effect on PC12 cell injury induced by Aβ_25–35_. Quercetin exhibited a similar protective effect on the H_2_O_2_-induced PC12 cell model [53]. Liu et al. reported that galantamine at a concentration of 10 µM significantly protected cells against Aβ_25–35_ neurotoxicity. At concentrations ranging from 0.01 to 100 µM, the protective effect of galantamine increased in a bell-shaped, dose-response manner [54]. This finding suggested that quercetin and galantamine might exert neuroprotective effects by different mechanisms. Yan showed that following Aβ_25–35_ treatment alone of PC12 cells, the activity and expression of AChE in PC12 cells were significantly increased. Therefore, quercetin possibly exerts a neuroprotective effect by inhibiting AChE activity and level [55].

As there was no significant difference in the protective effect of quercetin when the concentrations were 40 µM and 80 µM, 1–40 µM quercetin was applied in the following experiments.

### 2.11. Quercetin Reduced MDA Content and Increased GSH Levels in PC12 Cells

We measured the content of MDA (Figure 7C) and the activities of GSH (Figure 7D) by evaluating OS. MDA is an important index to evaluate membrane lipid peroxidation (MLP), whose level indicates MLP levels. The MDA released by PC12 cells exposed to 20 µM Aβ_25–35_ treatment for 24 h was significantly increased, while the release of MDA was inhibited by quercetin pretreatment. The effect was statistically significant relative to the model group (*p* < 0.01). GSH is a small molecular antioxidant that can scavenge free radicals and peroxides in the body. It has been identified to be a critical factor in maintaining the redox balance in cells. The GSH levels of the model group decreased significantly (*p* < 0.001). Compared with the model group, quercetin 1 µM pretreatment increased the GSH levels with no statistical difference (*p* > 0.05). However, the 10, 20, and 40 µM quercetin pretreatment of PC12 cells increased intracellular GSH levels in a dose-dependent manner. Based on the above findings, quercetin acts as an antioxidant to protect PC12 cells against oxidative damage [56].

### 2.12. Quercetin Reduced ROS Levels in Aβ_25–35_-Induced PC12 Cells

It has been reported that Aβ_25–35_ toxicity is induced through the production of ROS, and quercetin is able to quench ROS production [57]. Therefore, we studied whether quercetin blocked Aβ_25–35_-induced oxidative stress injury to PC12 cells. According to Figure 7E, the intracellular ROS contents of the Aβ_25–35_ model group increased dramatically (234.2%) relative to the normal control group. Furthermore, the corresponding intracellular ROS content decreased to 212.9%, 174.5%, 150.0%, and 129.7%, respectively, after quercetin pretreatment (1, 10, 20, and 40 µM, *p* < 0.05). ROS was an OS indicator related to the pathogenic mechanism of several neurodegenerative disorders. The current results suggested that quercetin could decrease ROS levels and exert neuroprotective effects against Aβ_25–35_-induced neurotoxicity. Zeng et al. reported the same results and views when studying the protective effects of artemisinin on PC12 cells against β-amyloid-induced apoptosis [58]. Quercetin increased cell viability and GSH levels and decreased the MDA content in PC12 cells induced by Aβ_25–35_, also suggesting that the antioxidant activity of quercetin contributes to its protective effects. However, Jiang et al. found that galantamine downregulated NOX4 expression to inhibit Aβ- mediated ROS accumulation and autophagy [59]. This further confirmed that quercetin and galantamine could decrease Aβ_25–35_-induced oxidative stress in different ways.

## 3. Materials and Methods

### 3.1. Materials

The amounts of 137 U/mg AChE (from *Electrophorus electricus*) and Aβ_25–35_ (purity ≥ 97%) were purchased from Sigma-Aldrich Co. (St. Louis, MO, USA). AChE was dissolved in phosphate-buffered saline (PBS) (0.1 M, pH 7.6) to prepare a 10 U/mL stock solution. Quercetin (purity ≥ 98.5%) was obtained from the National Institute for the Control of Pharmaceutical and Biological Products (Beijing, China). It was later dissolved in ethanol to form the 5.0 mM stock solution. Additionally, 5,5-Dithiobis-(2-nitrobenzoic acid) (DTNB), galantamine hydrobromide, 3-(4,5-dimethylthiazol-2-yl)-2,5-diphenyltetrazolium bromide (MTT), and acetylthiocholine iodide (ATCI) were obtained from Aladdin Chemistry Co., Ltd. (Shanghai, China). The fetal bovine serum (FBS) was obtained from Gibco (Carlsbad, CA, USA). Other culture chemicals such as DMEM, trypsin, and penicillin-streptomycin (P-S) were bought from Solarbio (Beijing, China). Malondialdehyde (MDA), ROS, and glutathione (GSH) were detected at the Jiancheng Bioengineering Institute (Nanjing, China) using a commercial kit. All remaining reagents were analytically pure, and fresh ultrapure water was used.

### 3.2. AChE Activity Assay

In vitro AChE activity assay was carried out according to Ellman’s approach after some modifications [60]. Briefly, 2 U/mL of AChE (50 µL, 3.4 × 10^−8^ M) was incubated with different concentrations of quercetin in a 3 mL sodium phosphate buffer reaction system (0.1 M, pH 7.6) for 30 min under 25 °C; next, 50 µL of the DTNB solution was introduced. Subsequently, 15 mM ATCI (50 µL) was added to initiate the catalyzing reaction. The spectrophotometer (UV-2450, Shimadzu, Kyoto, Japan) was later used to monitor the absorbance (OD) value at 412 nm and 10 s intervals for 200 s. Galantamine hydrobromide was adopted as a positive control. The AChE activity was defined to be 100%, whereas the IC_50_ value (quercetin content at the relative enzyme activity of 50%) was taken as inhibition of quercetin on AChE.

### 3.3. Inhibitory Type Kinetics Analysis

The inhibitory kinetics of quercetin on AChE were analyzed using the same method that was used to determine the enzyme activity. The concentration of the substrate ATCI (0.25 mM) was fixed, and when the concentration of quercetin was 0, 2.0, 3.2, 4.0, and 8.0 µM, the trend of the reaction rate with increasing AChE concentration was determined to evaluate the reversibility of inhibition. The AChE (0.57 nM) concentration was kept unchanged, and the quercetin reaction rate was measured at different concentrations (0, 4.0, 8.0, and 12.0 µM) with increasing ATCI concentration. The type of inhibition was determined using the Lineweaver-Burk plot in double reciprocal form.

The time–course tests were used to determine the enzyme inactivation rate constants and the kinetic process with different concentrations of quercetin as 1.5, 3.0, 6.0, and 15.0 µM. We determined enzyme activities at diverse contents every 3 min for 0–30 min and every 5 min for 30–70 min.

### 3.4. Fluorescence Spectrum Measurement

Hitachi Fluorescence Spectrophotometer (model F-7000; Tokyo, Japan) was used to measure the AChE fluorescence spectra with quercetin at three different temperatures (25, 31, and 37 °C) by controlling the sample cell temperature in a circulating water bath device. Next, 1 µL of 2.0 mM quercetin solution was added to 2.5 mL of AChE solution (0.17 µM) ten times successively. After each drop, the solution was mixed and left for 3 min to balance the solution. The fluorescence emission spectra at 290 to 450 nm were scanned at an excitation wavelength of 280 nm, with emission and excitation slot widths of 2.5 nm.

The fluorescence results were adjusted to eliminate the internal filtration effect caused by the ultraviolet absorption of quercetin according to equation (Equation (S1)) [35].

### 3.5. CD Spectra Measurement

The MOS 450 CD spectrometer (Bio-Logic, Claix, France) was used to measure the CD spectra for AChE solution at 190 to 250 nm under ambient temperature. CD spectral signals off free AChE solution (0.75 µM) and the quercetin–AChE composite system with different concentration ratios were collected by deducting buffer signals, and the content of the discrepant secondary structures was calculated. The method to analyze the content of specific secondary structures has been reported in our previous study [36].

### 3.6. Molecular Docking

The CDOCKER algorithm was used to simulate docking between quercetin and AChE using the Discovery Studio 4.5 software. The crystal structure of AChE (PDB ID: 4EY7) was retrieved from the RCSB Protein Data Bank (http://www.rcsb.org/pdb. Accessed on: 28 September 2021) [30]. Additionally, the three-dimensional (3D) structure of quercetin was downloaded from the PubChem online database (https://pubchem.ncbi.nlm.nih.gov/, Accessed on: 28 September 2021), and molecular optimization was performed to obtain the conformation with minimal energy. Before docking, the crystal structure of AChE was treated by depleting water molecules, adding hydrogen atoms, and adding polarity. After adding the CHARMm force field by algorithm, AChE was considered the receptor and quercetin the ligand. Hotspot number and docking tolerance were set at 100 and 0.25, respectively. According to the lowest CDOCKER interaction energy results, the best docking conformation was selected to study the interaction between quercetin and AChE.

### 3.7. MD Analysis

The MD simulation studies for the AChE–quercetin complex were conducted using the GROMACS 5.1.2 software under the Linux operating system and applying the method described by Ni [22]. AChE’s PDB file conformed to that of molecular docking. Furthermore, force fields of the general amber and AMBER99SB-ILDN proteins were introduced for charging quercetin and AChE, respectively. Acpype Server was used to process the quercetin topology file, and the total topology file was obtained by combining quercetin with AChE. The complex was placed in a dodecahedral box of a simulated simple point charge (SPC) water molecule model with an edge distance of 1 nm [51]. Furthermore, 16 Na^+^ and 6 Cl^−^ were introduced to neutralize the surface charges of the complex, followed by energy minimization and equilibration of the minimized system using NVT for 100 ps at 300 K and 1 bar. Finally, MD simulation was performed for 60 ns using the NPT ensemble.

### 3.8. Analysis of Combined Inhibition

The combined effect of quercetin and galantamine hydrobromide in inhibiting the AChE activity was analyzed using a dose-normalized isobologram [61]. Using the same method described in Section 3.2, the quercetin or galantamine hydrobromide dose was defined as 100% when it inhibited X% (X = 30, 50, and 70) activity of AChE alone. The dose of galantamine hydrobromide was fixed at 20%, 40%, 60%, and 80% of the corresponding inhibition rate, and the dose of quercetin was changed to achieve an identical inhibitory action. When the inhibition rate of enzyme activity was X%, the doses of the two components (*D*_1_ and *D*_2_) were considered as the X and Y axes, respectively, and the equivalent line maps of each combination were obtained. The combination index (*CI*) was used to quantitatively evaluate the combined effect of inhibiting X% enzyme activity:

(1) $$CI=\frac{{\left(D\right)}_{1}}{{\left(D_{X}\right)}_{1}}+\frac{{\left(D\right)}_{2}}{{\left(D_{X}\right)}_{2}}$$

*D_X_* refers to the dosage when quercetin and galantamine hydrobromide individually inhibit X% enzyme activity. *CI* < 0.9 stands for synergism, 0.9–1.1 indicates additive interaction, whereas >1.1 represents antagonism.

### 3.9. Cell Culture and Treatment

PC12 cells were procured from Nanjing KeyGen Biotech Co. Ltd. (Nanjing, China). The cells were maintained in high-glucose DMEM containing 10% fetal bovine serum (FBS) and 1% P-S and incubated under 5% CO_2_ and 37 °C. PC12 cells in the logarithmic phase were seeded in 96-well (approximately 2 × 10^4^/well) or 24-well (approximately 1 × 10^5^/well) culture plates, incubated for 24 h, followed by the replacement of the medium.

The cells were classified into six groups, namely, blank control (normal medium); Aβ_25–35_ model (20 µM Aβ_25–35_, serum-free DMEM medium, 24 h); and quercetin (pretreatment with 1, 10, 20, and 40 µM quercetin for 2 h, followed by another 24 h treatment using 20 µM Aβ_25–35_). Each group was provided with six duplicate wells based on different measurement indexes.

### 3.10. Quercetin-Induced Toxicity in PC12 Cells

Quercetin was dissolved in dimethylsulfoxide (DMSO) to prepare a 5.0 mM stock solution, diluting it using a serum-free medium until the required concentration was obtained. Subsequently, we inoculated PC12 cells in a 96-well culture plate for 24 h. After changing the culture medium, quercetin was added to the final concentrations of 0, 1, 10, 20, 40, and 80 µM. Six replicate wells were completed for each concentration group, followed by another 24 h culture. To determine the role of quercetin in cell viability, all wells were introduced with 5 mg/mL MTT solution (20 µL) and incubated for 4 h at 37 °C. Subsequently, 150 µL of DMSO was added to dissolve the resulting formazan crystals for 10 to 15 min, then measuring the OD value at 570 nm using a microplate reader (Varioskan LUX; Thermo Fisher Scientific, Waltham, MA, USA). The cell viability is represented as the proportion relative to the control group [55].

### 3.11. Role of Quercetin in Aβ_25–35_-Induced PC12 Cell Viability

PC12 cells were inoculated in 96-well plates for 24 h. After incubation according to the method described in Section 3.9 (pretreatment with 1, 10, 20, 40, and 80 µM quercetin), the MTT method was adopted to measure cell viability.

### 3.12. Determination of MDA, GSH, and ROS in PC12 Cells

For cell culture and grouping sample addition, please refer to Section 2.9. After incubation, the culture plate was centrifuged at 200× *g* for 5 min using a plate centrifuge, and the supernatant was discarded. Next, the cells were collected and broken by ultrasound and centrifuged at 3000 r/min at 4 °C for l0 min, and the supernatant was collected for subsequent assays. The OD was recorded at 532 and 405 nm, respectively, following the instructions mentioned in the manual for the MDA and GSH kits. Intracellular MDA content and GSH levels are expressed as percentages of the control.

PC12 cells (1 × 10^4^/well) were inoculated into a 96-well culture plate. After culture, the culture solution was sucked out, sterile PBS was added to each well to rinse gently twice for 3 min each time, and then the cells were treated according to the steps mentioned in the manual. Fluorescence intensities (ROS levels) were recorded at the emission and excitation wavelengths at 525 and 480 nm, respectively, using the microplate reader (Varioskan LUX). The intracellular ROS level is represented by the proportion relative to the control.

### 3.13. Statistical Analysis

All samples were tested in triplicate. Results are represented as mean ± standard deviation (SD). Origin 9.0 was adopted for data analyses using one-way analysis of variance (ANOVA), and *p* < 0.05 was considered statistically significant.

## 4. Conclusions

In summary, quercetin showed a mixed inhibitory effect on AChE, *K*_i,_ and α*K*_i_ being (6.17 ± 0.18) and (19.53 ± 0.65) µM, respectively. Quercetin can quench the fluorescence of AChE statically and interact with AChE via van der Waals forces and hydrogen bonding. The binding constant between them is in a 10^4^ L mol^−1^ order, and quercetin has only one binding site on the enzyme. After interaction with quercetin, the α-helix content of AChE increased, while that of the β-sheet content decreased, and the structure of AChE changed to a more compact conformation. Quercetin is combined with the PAS site of AChE and important amino acid residues (Asp74, Tyr124, Phe338, Tyr337, and Tyr341) by hydrophobic interaction, van der Waals forces as well as hydrogen bonding to form a stable complex. Furthermore, the interaction of quercetin with the PAS site induces allosterism of AChE, preventing the substrate from combining with the enzyme active site, thus inhibiting the enzyme. Meanwhile, quercetin extends to the AChE’s active center. It generates hydrogen bonds with Glu202 and Tyr133, further reducing the catalytic activity of AChE by destroying the stability of the hydrogen bond network in the active center of the enzyme. The results of the MD simulation show the increased stability of the quercetin-AChE complex, and complex formation reduces the flexibility of the molecules, making the structure more compact. Quercetin and galantamine interact with the amino acid residues at the AChE active center sites to achieve the synergistic inhibitory effect. Quercetin can increase the level of antioxidant GSH and decrease the content of lipid peroxidation end product MDA. Moreover, quercetin can scavenge Aβ_25–35_-mediated ROS levels in damaged PC12 cells, thus exerting neuroprotective effects. In conclusion, quercetin plays an anti-AD function by inhibiting AChE and protecting the neurons from oxidative stress injury. These results may provide scientific guidance for dietary prevention and treatment of AD with quercetin. Furthermore, it provides novel insights for applying quercetin in functional foods, anti-AD drug research, and developing new AChE inhibitors.

## Figures and Tables

**Table 1:** Inactivation rate constants for AChE with quercetin.

Quercetin (µM)	Inactivation Rate Constant ^1^ (×10^−4^ s^−1^)	Transition Free Energy Change ^2^ (kJ mol^−1^ s^−1^)
	*k*	
1.5	4.18 ± 0.06 ^d^	19.28
3.0	5.73 ± 0.03 ^c^	18.49
6.0	6.04 ± 0.08 ^b^	18.36
15.0	6.41 ± 0.05 ^a^	18.22

^1^*k* is the inactivation rate constant. Different letters “a–d” mean that the values of *k* were significantly different (*n* = 3, *p* < 0.05); ^2^ Transition free-energy change, ΔΔ*G*° = −*RT*ln*k*, where *k* is the time constant of the inactivation reaction.

**Table 2:** The quenching constants (*K*_SV_), binding constants (*K*_a_), and relative thermodynamic parameters for quercetin-AChE interaction under three temperature conditions.

*T* (°C)	*K*_SV_ (×10^4^ L mol^−1^)	*R* ^a^	*K*_a_ (×10^4^ L mol^−1^)	*R* ^b^	*n*	∆*H*° (kJ mol^−1^)	∆*G*° (kJ mol^−1^)	∆*S*° (J mol^−1^ K^−1^)
25	6.21 ± 0.08 ^a^	0.9983	5.52 ± 0.05 ^a^	0.9976	0.75 ± 0.04	−63.39 ± 0.07	−27.09 ± 0.03	-121.8 ± 0.06
31	4.81 ± 0.06 ^b^	0.9968	3.52 ± 0.03 ^b^	0.9983	0.69 ± 0.01		−26.37 ± 0.05	
37	3.33 ± 0.05 ^c^	0.9979	2.05 ± 0.02 ^c^	0.9975	0.67 ± 0.03		−25.64 ± 0.07	

*R* ^a^ indicates the correlation coefficient of the *K*_SV_ values; *R* ^b^ represents the correlation coefficient of the *K*_a_ values. The different letters “a–c” indicate a significant difference (*n* = 3, *p* < 0.05).

**Table 3:** Quercetin–AChE complex’s secondary structure levels at diverse molar ratios.

Molar Ratio (Quercetin):(AChE)	α-Helix (%)	β-Sheet (%)	β-Turn (%)	Random Coil (%)
0:1	34.35 ± 0.74 ^d^	18.62 ± 0.06 ^a^	19.18 ± 0.28 ^d^	28.02 ± 0.65 ^a^
2:1	37.13 ± 0.16 ^c^	17.31 ± 0.09 ^b^	20.15 ± 0.24 ^c^	25.41 ± 0.34 ^b^
6:1	38..47 ± 0.21 ^b^	16.20 ± 0.17 ^c^	21.62 ± 0.13 ^b^	23.71± 0.28 ^c^
10:1	41.51 ± 0.28 ^a^	15.16 ± 0.14 ^d^	23.14 ± 0.32 ^a^	20.19 ± 0.37 ^d^

The different letters (a–d) indicate a significant difference (*n* = 3, *p* < 0.05).

## Data Availability

The data in this study are available in the article.

## Supplementary Materials

The following supporting information can be downloaded at: https://www.mdpi.com/article/10.3390/molecules27227971/s1, Equations (S1)–(S8); Figure S1: Fluorescence spectra of quercetin at varying concentrations (pH 7.6, T = 25 °C). *c*(quercetin) = 0, 0.8, 4.8 and 8.0 μM, respectively; Figure S2: Docked conformer of donepezil (white) and original pose of co-crystallized donepezil (red).
