# Supplementary material for: Exploring the Inhibition of Quercetin on Acetylcholinesterase by Multispectroscopic and In Silico Approaches and Evaluation of Its Neuroprotective Effects on PC12 Cells
Source: Molecules. 2022 Nov 17;27(22):7971. doi: 10.3390/molecules27227971 (PMC9699400; doi:10.3390/molecules27227971)
Supplement: Supplementary file 1 [file molecules-27-07971-s001.zip › molecules-2033387-supplementary.pdf]

Supporting Information

Eqation S1

$$F_{\mathrm{c}} = F_{\mathrm{m}} e^{(A_1 + A_2)/2} \qquad \text{(S1)}$$

Where F_{c} and F_{m} represent fluorescence intensity values for correction and determination, respectively. A_1 and A_2 represent quercetin's absorbance levels at fluorescence excitation and emission wavelengths, respectively.

Equations S2 – S4

$$\frac{1}{v} = \frac{K_{\mathrm{m}}}{V_{\mathrm{max}}}(1 + \frac{[\mathrm{I}]}{K_{\mathrm{i}}})\frac{1}{[S]} + \frac{1}{V_{\mathrm{max}}}(1 + \frac{[\mathrm{I}]}{\alpha K_{\mathrm{i}}}) \qquad \text{(S2)}$$

$$\mathrm{Slop} = \frac{K_{\mathrm{m}}}{V_{\mathrm{max}}} + \frac{K_{\mathrm{m}}[\mathrm{I}]}{V_{\mathrm{max}} K_{\mathrm{i}}} \qquad \text{(S3)}$$

$$\text{Y-intercept} = \frac{1}{V_{\mathrm{max}}^{\mathrm{app}}} = \frac{1}{V_{\mathrm{max}}} + \frac{1}{\alpha K_{\mathrm{i}} V_{\mathrm{max}}}[\mathrm{I}] \qquad \text{(S4)}$$

In these formulas, [I] represent inhibitor content, whereas [S] stands for substrate content; ν and V_{max} represent enzymatic reaction rate and maximum catalytic reaction rate, respectively; K_{m} is the Michaelis−Menten constant; K_{i} and αK_{i} stand for the equilibrium constants of quercetin binding to the free AChE and AChE−substrate complex, and α is the apparent coefficient.

Equations S5

$$\frac{F_0}{F} = 1 + K_{\mathrm{SV}}[Q] = 1 + K_{\mathrm{q}}\tau_0[Q] \qquad \text{(S5)}$$

In the formula, F_0 stands for system fluorescence intensity with AChE alone, whereas F indicates that with quercetin added, respectively, and K_{SV} stands for the Stern–Volmer quenching constant, which represents fluorescence quenching efficiency, $[Q]$ denotes quercetin content, K_{q} indicates bimolecular quenching rate constant, whereas τ_0 denotes the average fluorophore lifetime, about 10^{-8} s.

Equations S6

$$\log\frac{F_0 - F}{F} = n\log K_a - n\log\frac{1}{[Q_t] - \frac{(F_0 - F)[P_t]}{F_0}} \qquad \text{(S6)}$$

$[Q_t]$ and $[P_t]$ denote quercetin and AChE concentrations, respectively. F_0 and F are the same as in Equation S 5.

Equations S7 – S8

$$\log K_a = -\frac{\Delta H°}{2.303RT} + \frac{\Delta S°}{2.303R} \qquad \text{(S7)}$$

$$\Delta G° = \Delta H° - T\Delta S° \qquad \text{(S8)}$$

where K_a and R suggest binding and gas (8.314 J mol^{-1} K^{-1}) constants, respectively. Temperature (T) was set to 25, 31, and 37 °C.

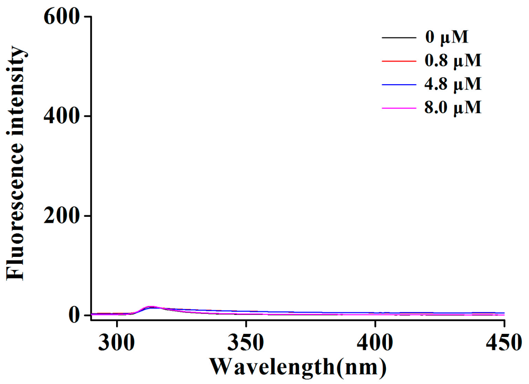

Figure S1. Fluorescence spectra of quercetin at varying concentrations (pH 7.6, T = 25 °C). c(quercetin) = 0, 0.8, 4.8 and 8.0 μM, respectively.

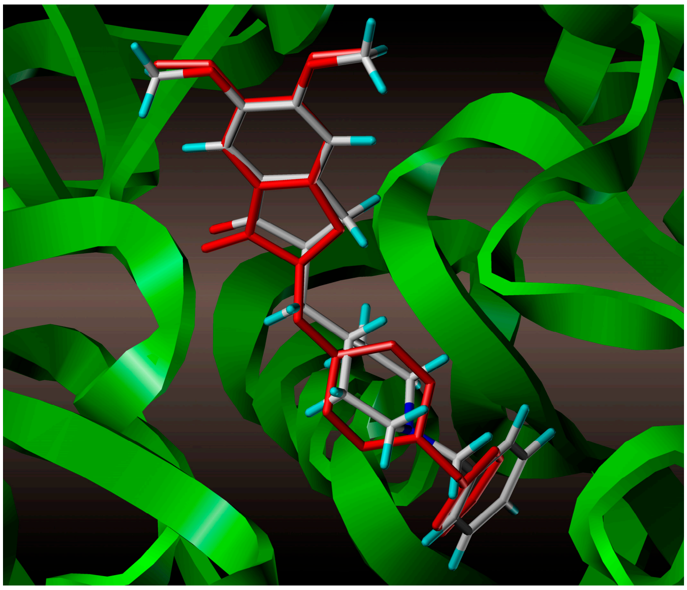

Figure S2. Docked conformer of donepezil (white) and original pose of co-crystallized donepezil (red).
